# Supplementary material for: No changes in parieto‐occipital alpha during neural phase locking to visual quasi‐periodic theta‐, alpha‐, and beta‐band stimulation
Source: Eur J Neurosci. 2018 Aug 3;48(7):2551–65. doi: 10.1111/ejn.13935 (PMC6220955; doi:10.1111/ejn.13935)
Supplement: Supplementary file 2 [file EJN-48-2551-s002.pdf]

EJN European Journal of Neuroscience FENS Federation of European Neuroscience Societies

No changes in parieto-occipital alpha during neural phase locking to visual quasi-periodic theta-, alpha-, and beta-band stimulation

Christian Keitel, Christopher Benwell, Gregor Thut and Joachim Gross

Review timeline:

Submission date:	25 November 2017
Editorial Decision:	23 January 2018
Revision received:	21 March 2018
Accepted:	10 April 2018

Editor: Heleen Slagter

1st Editorial Decision 23 January 2018

Dear Dr. Keitel,

Your manuscript was reviewed by external reviewers as well as by the Section Editor, Dr. Heleen Slagter, and ourselves.

As you can see, both reviewers thought that your study was well conducted, using state of the art and rigorous analysis and statistical testing. Both raised several helpful points that will make the manuscript even stronger and both categorized their comments as 'minor revisions'. Please address each of their points in the revised version and in particular, it would be very informative and help comparison to previous studies, if you also examine and discuss how alpha power lateralization was affected in rhythmic stimulation conditions vs. quasi-rhythmic conditions.

Thank you for submitting your work to EJN.

Best wishes,

Paul & John
co-Editors in Chief, EJN

Reviews:

Reviewer: 1 Rasa Gulbinaite (CERCO, Fance)

Comments to the Author
In this manuscript, Keitel et al. present re-analysis of the data published in their original study (PMID: 27867090). Here, they focused on the effects of bilateral band-limited visual stimulation in three different frequency bands (theta, 4-7 Hz; alpha, 8-13 Hz; and beta, 14-20 Hz) on alpha-band power and behavior in spatial attention task. The authors demonstrate that quasi-rhythmic stimulation across three different conditions neither did differentially affect alpha power lateralization nor behavior. The authors conclude that low-intensity quasi-rhythmic visual stimulation cannot be used to entrain endogenous alpha generators.
The manuscript is clearly written and addresses an important question. I have a few remarks and points of clarification:
1. As authors point out, most studies addressing the question of entrainment of endogenous brain rhythms using sensory stimuli have used rhythmic stimulation protocols and have reported effects on alpha power and/or behavior. Given that the authors aim to “set boundary conditions for when and how to expect effects of entrainment of alpha generators” (Abstract), comparison of the effects of quasi-rhythmic and purely rhythmic stimulation (10 Hz and 12 Hz) on alpha-band power seems to be logical and necessary. The two aforementioned rhythmic stimulation conditions are part of the original design (as noted in p. 8), and thus would be useful to see how alpha power lateralization was affected in rhythmic stimulation conditions vs. quasi-rhythmic conditions.
2. Relatedly, setting out boundary conditions for entrainment of endogenous rhythms implies parametrically testing how deviations from rhythmicity in the stimulus affects endogenous oscillators. Even though the original study was not design for this purpose, band-limited frequency fluctuations of the stimulus presumably contained periods of more rhythmic vs. less rhythmic stimulation. Comparing the effects on alpha power between these two extremes thus could be informative and would allow to delineate “boundary conditions for when and how to expect effects of entrainment of alpha generators”.
3. The authors propose that stronger increase in alpha-band power during alpha vs. theta or beta quasi-rhythmic stimulation would serve as evidence for entrainment of intrinsic alpha rhythms. This indeed is a valid criterion; however, the classical notion of resonance implies stronger responses to some stimulation frequencies than the others [e.g. when stimulating with wide-range of frequencies (Herrmann, 2001; PMID: 11355381) or using broad-band flicker stimuli (VanRullen & Macdonald, 2012, PMID: 22560609)]. Fully testing the presence of alpha resonance

would additionally require comparing the effects of quasi-rhythmic stimulation on theta- and beta-band power. That is quantitatively testing the results presented in Figure 1d (rightmost panel).
4. The authors “expected individual alpha frequencies, measured in the absence of stimulation, to be pulled towards the centre frequency of the alpha band stimuli during stimulation” (p. 21). Given the flat frequency spectrum in the alpha-band stimulation with no peak at the center frequency, it is not clear why this should be the case. Please expand the theoretical motivation behind this prediction.
5. In p. 14 the authors note “we subtracted left-lateralized (Attend-Left conditions) from right-lateralized (Attend-Right) alpha power topographies” , whereas Figure 3b says “Attend Left – Attend Right”.

Reviewer: 2 Joachim Lange (Heinrich Heine University, Germany)

Comments to the Author
Keitel and colleagues re-analyzed data from a previous study that used external visual stimulation to potentially entrain intrinsic neuronal oscillations in parieto-occipital cortex of humans, measured with EEG. In contrast to other studies that used strictly periodic stimuli, Keitel et al. used quasi-periodic stimulation, i.e., stimulation frequency varied within a given frequency band. Another difference to previous studies was that the quasi-periodic stimulation was non-informative for a behavioral task. The authors applied stimulation in the theta-, alpha-, and beta-band. With these different frequency bands, the authors wanted to test the hypothesis that only alpha-band stimulation should have an effect on alpha oscillations (which are believed to have a predominant role in the visual cortex).
In a nutshell, Keitel at. did not find any differences between between neuronal oscillations in response to stimulation within frequency bands, nor any specific effects of any of the stimulation conditions on individual alpha frequencies nor power.

The study is well conducted, using state of the art and rigorous analysis and statistical testing. The results are clear and provide important new insights to the field of entrainment of intrinsic neuronal oscillations. The study complies well with the aims and scopes as well as with the Guidelines of EJN.

I have no major concerns or comments regarding the analysis or results. A few minor issues:
1) The legend of Fig. 1 does not seem to adequately describe the figure (b: bottom/top should be left/right).

2) In the caption of Fig. 2, the authors refer the reader to the materials section and their previously published study. Yet, I believe that the figure should be self-explanatory and thus needs more information. For example, it is unclear from the legend what a “peri” block is? From the legend/figure it is also unclear whether the tasks in the two workflows are from different measurements? Finally, isn’t peak frequency determined from the power spectra, i.e., arrows should be different?

3) Methods section on Bayes factor (p 15): I am not sure whether a BF10<1 always indicates “inconclusive” evidence. As far as I understand, BF10 and BF01 are anti-correlated. Thus, BF10<1/3 also indicates that BF01>3, so that BF10<1/3 well provides evidence against the H1 and for the H0. If I understand correctly, the results are also interpreted this way. But the methods part seems to describe the fact a bit imprecisely.

4) In the introduction, it was not always fully clear to me why the authors wanted to use external stimulation that showed contrast and frequency modulations. For example, why not simply use low, but constant contrast stimuli? In this sense, the introduction might benefit from a clearer structure towards a clear hypothesis. Maybe also the first paragraph of the results section would fit better into the introduction to make things clearer?

5) It was not fully clear to me what the authors expected from the frequency modulation of external stimulation. I am not sure whether I missed something, but if there should be only one specific frequency (IAF) that can be entrained, the other stimulation frequencies are rather ineffective, leading to overall weaker effects of entrainment. Alternatively, the stimulation at all alpha frequencies might affect neuronal oscillations at these different frequencies. But then, the neuronal oscillation should quickly adapt to the external frequnecy, leading to quickly varying frequencies of neuronal oscillations in response to external stimulation. This in turn should also lower any potential effects of entrainment, as it is averaged across frequencies. Could the authors please elaborate on this in the introduction and/or discussion.

6) Finally, there might be several reasons for the observed non-effects. The authors did a good job in crtically discussing these limitations. I would like to add one or two points: Maybe any potential effect on neuronal oscillations is spatially limited so that the authors definition of sensors of interest is too wide, picking up many cortical sites that are not affected by the esternal stimulation at all, thus lowering a potential effect when averaging across several site. In addition, I like the authors point that potential effects might not necessarily be reflected in peak frequencies (see also Baumgarten et al. 2017, for similar discussion in the somatosensory domain). I believe this is an important point as many studies simply look at "peak frequencies" rather than studying all frequencies.

Authors’ Response 21 March 2018

Dear Dr Slagter,

Thank you very much for giving us the opportunity to revise our manuscript.
We hope our revision improves on the issues raised by you and the reviewers.
Please find more detailed information in the attached letter.

Kind regards,

EJN European Journal of Neuroscience FENS Federation of European Neuroscience Societies

Christian Keitel, on behalf of all co-authors.

Dear Editor and Reviewers,

Thank you very much for your positive evaluations and valuable comments. Please find below our point-by-point responses.
Note that major changes to the manuscript are kept in blue font to facilitate cross-referencing and a comparison with the previous version.
Further note that in the process of revising the manuscript we have also added an extra concluding paragraph at the end of the discussion for some final emphasis, moved a paragraph on alpha power imbalance from the Results to the Discussion and applied some minor changes elsewhere to improve comprehensibility (all marked blue).
We hope that you find our revisions agreeable.

Kind regards
Christian Keitel – on behalf of all authors

Response to Reviewers;

Editor
It would be very informative and help comparison to previous studies, if you also examine and discuss how alpha power lateralization was affected in rhythmic stimulation conditions vs. quasi-rhythmic conditions.
Re: As per your and Reviewer 1's request we have now added a parallel analysis thread including the rhythmic-stimulation condition in the Supplementary Materials. Please see our response to Reviewer 1, point 1 below for further details.

Reviewer 1
1. As authors point out, most studies addressing the question of entrainment of endogenous brain rhythms using sensory stimuli have used rhythmic stimulation protocols, and have reported effects on alpha power and/or behavior. Given that the authors aim to "set boundary conditions for when and how to expect effects of entrainment of alpha generators" (Abstract), comparison of the effects of quasi-rhythmic and purely rhythmic stimulation (10 Hz and 12 Hz) on alpha-band power seems to be logical and necessary. The two aforementioned rhythmic stimulation conditions are part of the original design (as noted in p. 8), and thus would be useful to see how alpha power lateralization was affected in rhythmic stimulation conditions vs. quasi-rhythmic conditions.
Re: This is an excellent point that we have considered extensively when putting together the manuscript. Originally, we decided against including the rhythmic condition because the visual stimulation differs on more than one dimension from the other conditions: In addition to covering different frequencies, it was also strictly rhythmic and the left stimulus (10 Hz) was consistently "slower" than the right (12 Hz). We were therefore hesitant to compare it directly with the other conditions. Statistical differences could not have been unambiguously attributed to one or the other factor manipulation. Also, having two distinct alpha-frequency stimuli presented simultaneously requires a different strategy for our second analysis path (see path 2 in Figure 2 & results in Figure 5). These caveats notwithstanding, we see that this point is potentially highly interesting and have included a comprehensive parallel analysis including this condition in the Supplementary Material. Please find all results in section "Analyses including the strictly-rhythmic stimulation condition", starting on page 7 and following. In brief, including this condition in the analysis did not yield qualitatively different results. We refer to this additional analysis and its results in the main text where applicable (see changes on page 6) but abstain from an in-depth interpretation based on the constraints mentioned earlier.

2. Relatedly, setting out boundary conditions for entrainment of endogenous rhythms implies parametrically testing how deviations from rhythmicity in the stimulus affects endogenous oscillators. Even though the original study was not design for this purpose, band-limited frequency fluctuations of the stimulus presumably contained periods of more rhythmic vs. less rhythmic stimulation. Comparing the effects on alpha power between these two extremes thus could be informative, and would allow to delineate "boundary conditions for when and how to expect effects of entrainment of alpha generators".
Re: We agree that this is an interesting point. Nonetheless, we feel like we would be stretching the data too far in such an analysis. As the reviewer correctly points out, the original study was not designed for this purpose. The frequency modulations that the stimuli show on each trial have not been controlled to the extent that we can guarantee a balanced continuum from more to less rhythmic sections. Please further note that in including the changes the reviewer suggested in point 1, we did not find any particular influence of constant-frequency stimulation on ongoing alpha. This suggests a similarly negative finding when contrasting more vs less rhythmic within quasi-rhythmic conditions. However, we will gladly consider your suggestion in the design of follow-up experiments.

3. The authors propose that stronger increase in alpha-band power during alpha vs. theta or beta quasi-rhythmic stimulation would serve as evidence for entrainment of intrinsic alpha rhythms. This indeed is a valid criterion; however, the classical notion of resonance implies stronger responses to some stimulation frequencies than the others [e.g. when stimulating with wide-range of frequencies (Herrmann, 2001; PMID:11355381) or using broad-band flicker stimuli (VanRullen & Macdonald, 2012, PMID: 22560609)]. Fully testing the presence of alpha resonance would additionally require comparing the effects of quasi-rhythmic stimulation on theta- and beta-band power. That is quantitatively testing the results presented in Figure 1d (rightmost panel).
Re: This is a very good point that we might not have stressed enough in the manuscript: Of course, resonance cannot simply be concluded from finding a response to a particular band-limited stimulus but only from a relatively greater response to that stimulus. However, we do not find an effect of alpha stimulation on alpha power in the first place, therefore precluding any further comparison

between frequency bands. We have addressed this issue by revising the following sections:
“We further tested whether intrinsic alpha resonates during alpha-band stimulation (Fedotchev et al., 1990; Schwab et al., 2006; Spiegler et al., 2011), i.e. shows relatively greater power increase than theta power during theta-band stimulation and beta power during beta-band stimulation.” – at the end of the introduction (page 5), in conjunction with changes made regarding Reviewer 2’s comment 4.
“Note that the absence of an effect of alpha-band stimulation on alpha power precluded further dedicated analyses of an alpha resonance effect, i.e. a greater increase in alpha power during alpha band stimulation than in theta power during theta-band or beta-power during beta-band stimulation. Although our stimulation may have produced increased theta power (see Figure 1d, right-most panel), we did not find a similar effect in the alpha band to compare this increase to.” – at the end of Results section “No evidence for an influence of visual stimulation frequency on alpha power”
“Alpha resonance, in turn, should have expressed as a greater increase in alpha power during stimulation within the IAF range (Fedotchev et al., 1990; Schwab et al., 2006) than theta and beta power during theta- and beta-band stimulation, respectively.” - second paragraph of the Discussion section “No evidence for entrainment of the intrinsic visual alpha rhythm by quasi-periodic stimuli”
Note that if we conduct the analysis suggested in your comment we do find effects of *theta* band stimulation as is already visible in Figure 1d. The analysis was conducted on power spectra of all three quasi-periodic stimulation conditions collapsed across Attend-Left and Attend-Right. The test statistic was a one-way ANOVA with the factor of stimulation frequency (theta, alpha or beta band) embedded in a permutation procedure (N = 5000) that clustered across the two dimensions EEG channels (using above described neighbourhood structure) and frequencies (2 – 20 Hz, in steps of 0.5 Hz), as implemented in the fieldtrip function ft_freqstatistics using the ‘montecarlo’ method and the ‘depsamplesFunivariate’ statistic). One effect emerged at occipital sites bilaterally (F_{sum} = 704.266, P = 0.033) and was indicative of an increase in power in the theta compared to all other conditions from 4 – 6 Hz (see Figure below). Notably the topography of this effect is highly similar to scalp maps that show maximum EEG-stimulus coupling in Keitel et al. 2017 (PMID: 27867090) and thus likely indicates the response to the stimulus. Another effect (F_{sum} = 1493.582, P = 0.005) emerged at right fronto-central sites and indicated systematically lower power in the 15 – 20 Hz band (again, during theta band stimulation). Although interesting, this effect (beta suppression during theta-band stimulation) is currently difficult to interpret.

We have not included these results as of now because we think they would distract from our main focus, the alpha rhythm.

4. The authors “expected individual alpha frequencies, measured in the absence of stimulation, to be pulled towards the centre frequency of the alpha band stimuli during stimulation” (p. 21). Given the flat frequency spectrum in the alpha-band stimulation with no peak at the center frequency, it is not clear why this should be the case. Please expand the theoretical motivation behind this prediction.
Re: We agree that the flat stimulus energy spectra as depicted in Figure 1d invite this criticism. Two factors are at play here: 1) Note that we re-used these from the earlier Keitel et al (2017). Thanks to your comment we noticed a minor flaw in this depiction for the present purpose: In the original analysis, EEG power spectra were calculated using the multi-taper approach in FieldTrip. We included the stimulus time series as data channels to facilitate the coherence approach in Keitel et al. (2017), giving them the characteristic flat-top shape spectral power distribution. Repeating the spectral analysis without tapering produced a more accurate description of the spectral content of the stimulation that does actually peak at 10.5 Hz for alpha-band stimulation (compare original figure, left, with new profile, centre, and blow-up, right). The blow-up further shows that stimulus power drops by ~5 dB (i.e. to about 30% of the maximum) towards the limits of the stimulated frequency band. 2) Power spectra have been re-scaled to fit the depiction of the EEG power spectra for illustrative purposes therefore additionally flattening the top visually. We have adapted all stimulus profiles in Figure 1d accordingly and added information on the scale of stimulus power to the figure caption.

5. In p. 14 the authors note “we subtracted left-lateralized (Attend-Left conditions) from right-lateralized (Attend-Right) alpha power topographies”, whereas Figure 3b says “Attend Left – Attend Right”.
Re: Thanks for catching this inconsistency. The figure caption is accurate. We have corrected the text accordingly.

Reviewer 2
1. The legend of Fig. 1 does not seem to adequately describe the figure (b: bottom/top should be left/right).
Re: Thank you for paying attention to the details. The legend has been corrected accordingly.

2. In the caption of Fig. 2, the authors refer the reader to the materials section and their previously published study. Yet, I believe that the figure should be self-explanatory and thus needs more information. For example, it is unclear from the legend what a “peri” block is? From the legend/figure it is also unclear whether the tasks in the two workflows are from different measurements? Finally, isn’t peak frequency determined from the power spectra, i.e., arrows should be different?
Re: To address your points we have taken the following steps: We have extended the caption for the Figure to be more self-explanatory. In addition to the Figure itself we now mention the meaning of ‘peri’ in the legend. Lastly, we renamed some of the boxes to avoid confusion regarding the arrows.

3. Methods section on Bayes factor (p 15): I am not sure whether a BF10<1 always indicates “inconclusive” evidence. As far as I understand, BF10 and BF01 are anti-correlated. Thus, BF10<1/3 also indicates that BF01>3, so that BF10<1/3 well provides evidence against the H1 and for the H0. If I understand correctly, the results are also interpreted this way. But the methods part seems to describe the fact a bit imprecisely
Re: We agree with the reviewer: BF01 = 1 / BF10 as stated in the manuscript. However, there might be a slight misunderstanding here

because we also state that "[…] the corresponding Bayes factor (called BF10) shows evidence for H1 (compared to H0) if it exceeds a value of 3, and no evidence if BF10 < 1 […]" which is correct. And at the end of the paragraph we add that: "For BF10 and BF01 values < 1 are taken as inconclusive evidence for either hypothesis" which seems to be what the reviewer is aiming at. We have added "[…] no evidence for H1 […]" (see first quote) to further clarify this point.

4. In the introduction, it was not always fully clear to me why the authors wanted to use external stimulation that showed contrast and frequency modulations. For example, why not simply use low, but constant contrast stimuli? In this sense, the introduction might benefit from a clearer structure towards a clear hypothesis. Maybe also the first paragraph of the results section would fit better into the introduction to make things clearer?
Re: Please note first that this report is based on an exploratory analysis of a previously recorded dataset to study brain responses to quasi-rhythmic visual stimulation (see Keitel et al. 2017, Neuroimage, as cited in the manuscript). The stimulation was originally designed for that purpose. However, we found that the stimulation notwithstanding clear alpha peaks in power spectra allowed a dedicated analysis of this neural phenomenon. We already mention the exploratory nature of this analysis and that the data is derived from a paradigm not designed for this particular purpose throughout the manuscript. We have now further clarified in the abstract that this investigation is a re-analysis of an existing dataset.
That said the quasi-periodic contrast manipulation is absolutely vital for driving continuous brain responses in the first place. Conceptually, it is this manipulation that should give rise to putative entrainment phenomena. Methodologically, it forms the basis of our frequency modulation – it is the frequency of the contrast changes that is being modulated. We have clarified this in the intro by revising the description of the stimulation:
"In the present report, we tested whether quasi-periodic contrast modulations of low-intensity visual input produce effects consistent with an entrainment of endogenous alpha rhythms."
According to your suggestion we have also moved a modified version of the first paragraph of the Results section into the Introduction (see pages 4 & 5).

5. It was not fully clear to me what the authors expected from the frequency modulation of external stimulation. I am not sure whether I missed something, but if there should be only one specific frequency (IAF) that can be entrained, the other stimulation frequencies are rather ineffective, leading to overall weaker effects of entrainment. Alternatively, the stimulation at all alpha frequencies might affect neuronal oscillations at these different frequencies. But then, the neuronal oscillation should quickly adapt to the external frequency, leading to quickly varying frequencies of neuronal oscillations in response to external stimulation. This in turn should also lower any potential effects of entrainment, as it is averaged across frequencies. Could the authors please elaborate on this in the introduction and/or discussion?
Re: Thank you for pointing this out. We absolutely agree with the reviewer's first statement ("[…] if there should be only one frequency …"). In fact, we think that we have covered this point extensively in section "Current results suggest boundary conditions …" in the Discussion section. We have revised a section of the introduction to further accommodate your request as follows:
"To date, most studies into the entrainment of endogenous alpha have indeed employed high-intensity, strictly periodic stimulation that was task-relevant: The detection of a probe stimulus depended on the relative phase of the entraining stimulation, i.e. stimulus phase generated predictions about the occurrence of the target (Mathewson et al., 2012; de Graaf et al., 2013; Spaak et al., 2014; Notbohm & Herrmann, 2016). However, the limiting factors under which entrainment of endogenous alpha occurs or ceases have yet to be examined. This will be necessary in order to comprehensively characterise it as a fundamental neural process that may contribute to visual perception. To date, it remains an open question whether alpha entrainment occurs automatically in natural vision because continuous, ecologically valid stimulation is quasi-periodic at best, does not need to be of high contrast and does not necessarily fall within the alpha frequency range (Kayser et al., 2003; Blake & Lee, 2005), e.g. lip movements during speech (Chandrasekaran et al., 2009; Park et al., 2016)."
Hopefully this, in connection with the following paragraph in the introduction, clarifies why our stimulation is an interesting probe for alpha entrainment processes. As for the second part of your comment ("Alternatively …") we are not exactly sure what the reviewer refers to with "effects of entrainment averaged across frequencies". Should alpha generators quickly adapt their phase to quasi-rhythmic stimulation online then we would expect the pull-effect of individual alpha frequencies towards the centre frequency of the alpha-band stimulation (10.5 Hz). We tested for this effect but, based on our data, could not substantiate it. In our opinion this issue has thus been covered by the corresponding results and the last paragraph on page 23 in the Discussion section.

6. Finally, there might be several reasons for the observed non-effects. The authors did a good job in critically discussing these limitations. I would like to add one or two points: Maybe any potential effect on neuronal oscillations is spatially limited so that the author's definition of sensors of interest is too wide, picking up many cortical sites that are not affected by the external stimulation at all, thus lowering a potential effect when averaging across several site. In addition, I like the authors point that potential effects might not necessarily be reflected in peak frequencies (see also Baumgarten et al. 2017, for similar discussion in the somatosensory domain). I believe this is an important point as many studies simply look at "peak frequencies" rather than studying all frequencies.
Re: To incorporate your concerns we have adapted the Discussion. Specifically, we now suggest looking into individually source-reconstructed data for maximal spatial resolution to tease apart contributing cortical generators. Also we extended on the argument of looking beyond peak frequencies.
"Note that these effects may be small and escape the here achieved spectral resolution (0.5 Hz). They may also depend non-linearly on the difference between IAF and stimulation centre frequency, such that participants with IAFs further removed from 10.5 Hz may be less prone to experience a pull effect. Further, considering that our stimulation occurred within a band around but not at the centre frequency, may have weakened a pull effect additionally. Lastly, work on the role of intrinsic rhythms in tactile perception has led to

the argument that focussing on individual peak frequencies only may neglect effects on rhythmic activity beyond the peak (Baumgarten et al. 2017). Also note that the EEG is likely dominated by a strong parieto-occipital alpha generator. However recent research suggests more than one concurrent alpha rhythmic ‘modes’ involved in visual processing (Keitel & Gross, 2016; Barzegaran et al., 2017). It is therefore possible that our stimulation failed to influence the dominant rhythm but affected a less prominent alpha component that is more difficult to observe given the limited spatial resolution of EEG recordings. Dedicated source reconstructions in future MEG experiments may shed further light on this issue.”
